# Temporal Stability of Nasal Tip Rotation and Projection Following Rhinoplasty Using a Novel Integrated Pentagonal Tip Support Technique

**DOI:** 10.3390/medicina62061060

**Published:** 2026-05-30

**Authors:** Şamil Şahin, Burak Erkmen, Mehmet Nuri Elgörmüş, Yeşim Esen Yiğit Koçer, Yaşar Kemal Duymaz, İbrahim Engin Çekin

**Affiliations:** 1 Department of Otolaryngology, Private ENT Practice, 34380 Istanbul, Turkey; drsamilsahin@gmail.com (Ş.Ş.); drburakerkmen@gmail.com (B.E.); 2 Department of Otolaryngology, Atlas University, 34408 Istanbul, Turkey; 3 Department of Otolaryngology, Umraniye Research and Training Hospital, 34764 Istanbul, Turkey; dryesimesenyigit@gmail.com; 4 Department of Otolaryngology, Sultan Abdulhamid Han Research and Training Hospital, 34668 Istanbul, Turkey; dryasarkemalduymaz@gmail.com (Y.K.D.); iecekin@yahoo.com (İ.E.Ç.)

**Keywords:** structural rhinoplasty, nasal tip stability, PTST, nasal tip projection, nasal tip rotation

## Abstract

*Background and Objectives:* Achieving long-term stability of nasal tip rotation and projection remains a challenge in rhinoplasty because of postoperative remodeling processes such as edema resolution, soft tissue redraping, and scar maturation. This study evaluated the temporal changes in nasal tip rotation and projection following rhinoplasty using the Pentagonal Tip Support Technique (PTST). *Materials and Methods*: A retrospective analysis was conducted on patients undergoing primary rhinoplasty using PTST, with standardized photographic assessments performed intraoperatively and at 6 and 12 months postoperatively. Nasal tip rotation and projection were quantified using the nasolabial angle and Goode ratio, respectively, and temporal changes were analyzed across postoperative intervals. *Results:* Both parameters demonstrated statistically significant reductions over time. However, the majority of changes occurred within the first 6 months, whereas later follow-up intervals demonstrated smaller changes and a reduced rate of change. Similar temporal patterns were observed across different skin types, and skin type was not significantly associated with late postoperative changes in rotation or projection. *Conclusions*: The findings of the present study suggest that PTST is associated with a temporal pattern characterized by greater early postoperative changes followed by smaller interval changes during later follow-up. These findings may reflect postoperative healing dynamics and structural support behavior following rhinoplasty. However, further prospective comparative studies are needed to better define the clinical behavior of PTST over extended follow-up periods relative to established structural rhinoplasty techniques.

## 1. Introduction

Rhinoplasty is a key procedure in facial plastic surgery, yet achieving consistent and stable outcomes remains a significant challenge [1]. Nasal tip rotation and projection are fundamental determinants of nasal aesthetics and overall facial harmony, and even minor postoperative changes can significantly influence surgical outcomes [2]. However, the nasal tip is exposed to postoperative processes such as edema resolution, soft tissue redraping, and scar maturation, all of which may lead to progressive alterations in tip position over time [3]. Accordingly, achieving long-term stability in nasal tip rotation and projection continues to represent a major challenge in rhinoplasty.

Various surgical techniques have been developed to improve nasal tip support and enhance the stability of rotation and projection, including columellar strut grafts and septal extension grafts [4]. These approaches aim to reinforce the structural framework of the nasal tip and are widely adopted in contemporary rhinoplasty practice. Despite their widespread use and demonstrated capacity to provide structural support, achieving consistent long-term stability and predictability remains challenging [5,6]. In particular, the extent and timing of postoperative changes in nasal tip position are not fully understood, and the temporal pattern of rotation and projection changes has yet to be clearly characterized.

In response to these challenges, a novel grafting strategy aimed at supporting nasal tip stability is introduced. The Pentagonal Tip Support Technique (PTST) is based on a structurally integrated graft design intended to provide support to the nasal tip complex. Rather than relying solely on isolated structural reinforcement, the technique incorporates a geometric configuration and multi-point fixation strategy designed to achieve integrated support between the septal framework and tip-supporting structures. The present study aimed to evaluate postoperative changes in nasal tip rotation and projection and to assess the temporal behavior of these parameters following application of the PTST.

## 2. Materials and Methods

This study was conducted as a retrospective observational analysis including patients who underwent primary rhinoplasty using the PTST. Patients who underwent revision rhinoplasty and patients requiring additional costal cartilage harvesting and augmentation procedures were excluded to ensure a homogeneous study population. In addition, cases without complete standardized photographic documentation at all predefined time points were not included in the analysis. Ethical approval was obtained from Istanbul Medipol University on 19 June 2025 (Decision Number: 749).

All measurements were performed using digital image analysis software (ImageJ (version 1.54r), National Institutes of Health, Bethesda, MD, USA). Standardized lateral photographs were obtained at three time points: intraoperatively and at 6 and 12 months postoperatively. Photographs were obtained under standardized conditions, including a fixed camera distance, consistent head positioning, and a neutral facial expression. The nasolabial angle was defined as the angle between the columella and the upper lip, and the Goode ratio was calculated as the ratio of nasal tip projection to nasal length.

Changes in nasal tip rotation and projection were calculated as the differences between measurements obtained at predefined time intervals (0–6 months, 6–12 months, and 0–12 months). Paired comparisons were performed to evaluate temporal changes. In addition, the magnitude of change between the early (0–6 months) and late (6–12 months) postoperative periods was compared by analyzing the corresponding interval differences.

To assess whether postoperative changes between 6 and 12 months were influenced by patient characteristics, changes in the nasolabial angle and Goode ratio during this interval were modeled as dependent variables, with age and skin type included as independent variables.

### 2.1. Surgical Technique

**Figure 1:**
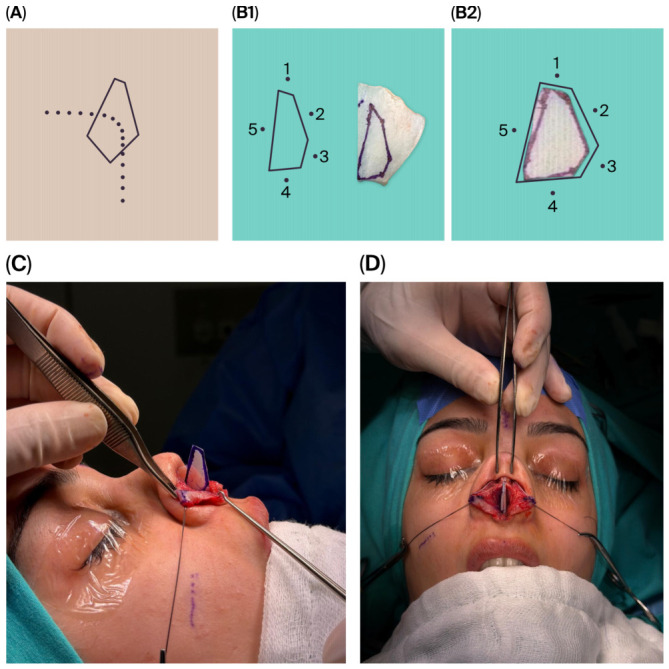
Surgical steps of the Pentagonal Tip Support Technique (PTST). (**A**) Geometric design of the asymmetric pentagonal graft, demonstrating a broad caudal base and a tapered cephalic extension. The dotted lines illustrate the intended directional support and vector of tip stabilization. (**B1**,**B2**) Translation of the geometric design into the final cartilage graft. (**B1**) Conceptual planning and carving of the graft based on the predefined geometric configuration. (**B2**) Final conformity of the sculpted graft, demonstrating alignment with the planned design and structural consistency. (**C**) Intraoperative midline placement of the graft at the level of the anterior septal angle, establishing structural continuity with the septum. (**D**) Final intraoperative configuration of the nasal tip complex, demonstrating integrated stabilization of the graft with the septum and domal structures, forming a unified support framework.

All procedures were performed by a single surgeon using a standardized open rhinoplasty approach under general anesthesia. After elevation of the skin–soft tissue envelope, the lower lateral cartilages and septal framework were fully exposed, and cartilage graft material was obtained from the septum or, when insufficient, from costal cartilage based on intraoperative assessment and structural requirements. The graft design was based on an asymmetric pentagonal configuration with a broad caudal base and a progressively narrowing cephalic extension (Figure 1A). The graft was then sculpted according to this predefined geometry, ensuring precise correspondence between the planned design and the final cartilage form (Figure 1B). The graft was positioned in the midline at the level of the anterior septal angle, establishing structural continuity with the septum and supporting the anterior septal segment (Figure 1C).

Fixation to the septum was achieved using two loop sutures placed at the anterior and posterior aspects of the graft with 5-0 polydioxanone (PDS) and a round needle, preventing both anterior and posterior displacement. In addition, at least three half-mattress sutures (5-0 PDS) were placed along the graft–septum interface to eliminate interfacial dead space, ensure close adaptation, and promote structural integration between the graft and septum, with the aim of improving positional stability and minimizing the potential for lateral displacement. Following septal fixation, the membranous septum and interdomal ligament complex were reconstructed, and the graft was further stabilized posteriorly at the domal level. Domal stabilization was achieved using a loop suture technique in which a transdomal suture was passed through one dome, then through the graft, and subsequently through the contralateral dome in a reverse direction to form a loop configuration, anchoring both domes to each other and to the posterior aspect of the graft, thereby creating an integrated tip support configuration.

The broad caudal portion of the graft was designed to provide a stable anchoring interface with the septum, while the tapered cephalic extension advances toward the domal region, with the intention of supporting the nasal tip without excessive widening. To further enhance stability and symmetry, a columellar strut–like graft was placed between the medial crura in continuity with the modified extension graft, and the medial crura were approximated and stabilized with 6-0 PDS sutures passed through both grafts. Domal stabilization was performed in a staged manner, with initial domal approximation sutures placed using 5-0 PDS, followed by final configuration sutures using 6-0 PDS. In addition, the soft tissue complex between the medial crura was excised to prevent widening at the footplate level and to maintain a more compact tip configuration. Nasal tip rotation and projection were adjusted intraoperatively by modifying the spatial relationship between the graft, septum, and domal complex, and the final configuration was determined based on intraoperative aesthetic assessment (Figure 1D).

### 2.2. Statistical Analysis

Statistical analyses were performed using Jamovi software (version 2.7.17; the Jamovi project, Sydney, Australia). Continuous variables were expressed as mean ± standard deviation, and categorical variables as frequencies and percentages. The normality of data distribution was evaluated using the Shapiro–Wilk test. As the data did not meet the normality assumption, non-parametric tests were employed. The Friedman test was used to compare nasolabial angle and Goode ratio measurements across the three follow-up time points (intraoperative, 6 months, and 12 months), with the Durbin–Conover procedure applied for pairwise post hoc comparisons with adjustment for multiple testing. A post hoc power analysis was performed using GPower 3.1. Since GPower does not include a dedicated module for the Friedman test, the analysis was conducted for its parametric counterpart (repeated-measures ANOVA) with three within-subject time points, and the result was adjusted using the asymptotic relative efficiency (ARE) of the Friedman test (ARE = 0.955). Assuming a medium effect size (Cohen’s f = 0.25), α = 0.05, and a correlation of 0.5 among repeated measures, the parametric analysis yielded a power of 0.999 with *n* = 84. After ARE adjustment, the estimated power for the Friedman test was approximately 0.954, suggesting that the study had sufficient statistical power to detect temporal changes. Multiple linear regression analysis was performed to assess the effects of age and skin type on the changes in nasolabial angle and Goode ratio during the 6–12 month interval. Gender was not included as a predictor due to the markedly imbalanced distribution in our cohort (95.2% female vs. 4.8% male), which may limit the reliability of sex-based inferences because of insufficient statistical power. Model assumptions were verified prior to interpretation. A *p* value of less than 0.05 was considered statistically significant.

## 3. Results

A total of 84 patients who underwent rhinoplasty using the PTST were included in the study. The mean age was 28.0 ± 5.98 years. Of the patients, 80 (95.2%) were female and 4 (4.8%) were male. Skin type distribution was as follows: normal in 36 patients (42.9%), thick in 33 patients (39.3%), and thin in 15 patients (17.9%) (Table 1).

**Figure 2:**
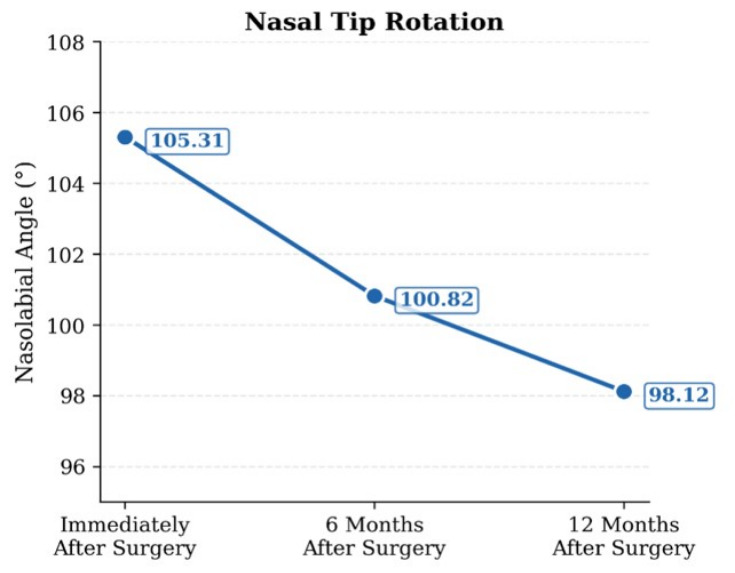
Temporal changes in nasal tip rotation (nasolabial angle) during the 12-month postoperative follow-up period (*n* = 84).

The nasolabial angle showed a decrease over time. The mean value was 105.31° ± 2.92° intraoperatively, 100.82° ± 2.77° at 6 months, and 98.12° ± 2.84° at 12 months. The mean decrease was 4.49° ± 1.31° for the 0–6 month interval, 2.70° ± 0.53° for the 6–12 month interval, and 7.19° ± 1.44° for the 0–12 month interval. The Friedman test demonstrated a statistically significant temporal change across follow-up time points, and post hoc pairwise comparisons revealed significant differences between all intervals (all *p* < 0.001) (Table 2, Figure 2)**.**

**Figure 3:**
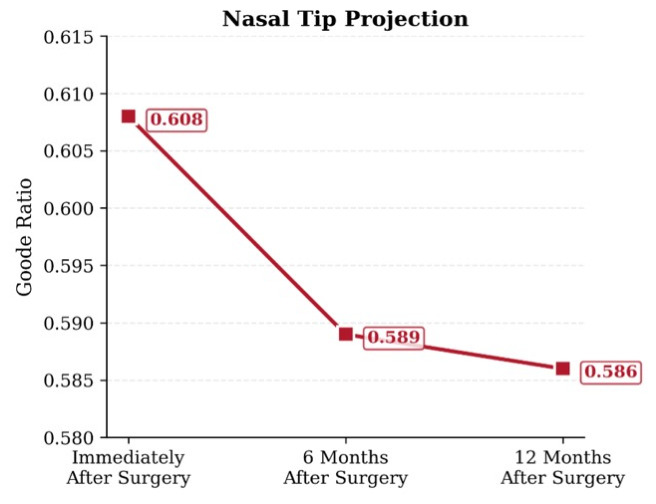
Postoperative changes in nasal tip projection (Goode ratio) over the 12-month follow-up period (*n* = 84).

The Goode ratio also showed a decrease over time. The mean value was 0.608 ± 0.022 intraoperatively, 0.589 ± 0.022 at 6 months, and 0.586 ± 0.022 at 12 months. The mean decrease was 0.019 ± 0.010 for the 0–6 month interval, 0.003 ± 0.002 for the 6–12 month interval, and 0.022 ± 0.010 for the 0–12 month interval. The Friedman test demonstrated a statistically significant temporal change across follow-up time points, and post hoc pairwise comparisons revealed significant differences between all intervals (all *p* < 0.001) (Table 3, Figure 3).

The magnitude of change between the 0–6 month and 6–12 month intervals was compared. The decrease in nasolabial angle was greater during the 0–6 month interval (4.49° ± 1.31°) than during the 6–12 month interval (2.70° ± 0.53°). Similarly, the decrease in the Goode ratio was greater during the first interval (0.019 ± 0.010) compared with the later interval (0.003 ± 0.002). Post hoc pairwise comparisons showed statistically significant differences between the postoperative intervals for both parameters (all *p* < 0.001) (Table 4).

**Figure 4:**
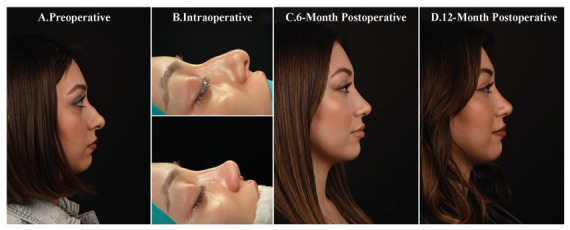
Representative clinical photographs of Patient 1 following PTST rhinoplasty: (**A**) preoperative view; (**B**) intraoperative views before and after PTST application; (**C**) 6-month postoperative view; (**D**) 12-month postoperative view.

**Figure 5:**
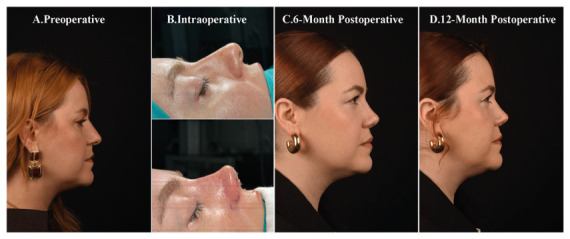
Representative clinical photographs of Patient 2 following PTST rhinoplasty: (**A**) preoperative view; (**B**) intraoperative views before and after PTST application; (**C**) 6-month postoperative view; (**D**) 12-month postoperative view.

**Figure 6:**
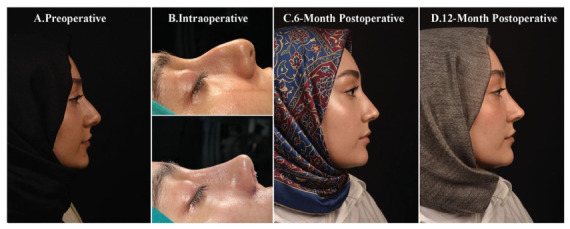
Representative clinical photographs of Patient 3 following PTST rhinoplasty: (**A**) preoperative view; (**B**) intraoperative views before and after PTST application; (**C**) 6-month postoperative view; (**D**) 12-month postoperative view.

Age and skin type were evaluated as predictors of nasolabial angle change between 6 and 12 months. The model was not statistically significant (F(3,80) = 0.320, *p* = 0.811, R^2^ = 0.012), and none of the variables showed a significant association (Table 5). A similar analysis was performed for the Goode ratio change between 6 and 12 months. This model was also not statistically significant (F(3,80) = 0.201, *p* = 0.895, R^2^ = 0.008), and no significant associations were identified (Table 6). No major intraoperative or postoperative complications related to the PTST were observed during the follow-up period. Representative preoperative, intraoperative, and postoperative clinical photographs demonstrating temporal changes in nasal tip rotation and projection following PTST are presented in Figure 4, Figure 5 and Figure 6.

## 4. Discussion

The present study evaluated the temporal behavior of nasal tip rotation and projection following rhinoplasty using the PTST. Both the nasolabial angle and the Goode ratio demonstrated statistically significant decreases over time; however, these changes should be interpreted within the context of normal postoperative healing. Minor reductions during the first 6 months are expected due to the resolution of edema and soft tissue redraping and do not necessarily indicate loss of structural support. During this period, the magnitude of change remained relatively limited. Although a further decrease was observed between 6 and 12 months, the rate of change was significantly lower compared to the early postoperative period. This pattern represented the main finding of the study. The observed deceleration in change suggests that postoperative changes become less pronounced after the initial healing phase. Rather than indicating structural weakening, these findings may reflect ongoing physiological remodeling following rhinoplasty. Overall, the present findings suggest a temporal pattern characterized by greater early postoperative change followed by smaller interval changes during later follow-up.

Changes in the nasolabial angle provide insight into the temporal behavior of nasal tip rotation following surgery [7,8]. In the present study, although a decrease in rotation was observed over time, the most notable finding was the reduction in the rate of change after the early postoperative period. While the majority of rotational adjustments occurred within the first 6 months, the subsequent decline between 6 and 12 months was smaller [9]. The early decrease in nasal tip rotation may be explained by resolution of edema, soft tissue redraping, and scar maturation, which are recognized components of postoperative healing dynamics in rhinoplasty [10,11]. This observation is clinically relevant, as nasal tip rotation plays an important role in nasal aesthetics and overall facial harmony [12]. Even subtle postoperative changes over time in rotation may influence patient perception, particularly in individuals who initially perceive their postoperative appearance as satisfactory but later notice changes in nasal tip position [13]. From a surgical perspective, uncertainty regarding the extent of postoperative rotational change may contribute to a tendency toward intraoperative over-rotation in some cases. However, this approach may also carry the risk of an over-rotated appearance if the anticipated degree of postoperative decrease does not occur. Conversely, in patients desiring greater rotation, postoperative reduction over time may contribute to a relatively under-rotated appearance. The findings of the present study suggest that PTST is associated with a temporal pattern characterized by greater early postoperative change followed by smaller interval changes during later follow-up.

Nasal tip projection demonstrated a decrease predominantly within the first 6 months following surgery. The magnitude of this early change remained relatively limited and is consistent with expected postoperative tissue remodeling, including edema resolution and soft tissue redraping [14,15]. Beyond the sixth postoperative month, projection demonstrated smaller interval changes and a reduced rate of change compared to the initial postoperative period. This transition from early adjustment to a slower rate of change represented the main observation regarding projection dynamics [16]. From a clinical perspective, preservation of nasal tip projection is important for maintaining nasal definition and facial balance, and progressive loss over time is generally considered undesirable [17,18]. Anticipating a certain degree of postoperative reduction, surgeons may tend to create slightly increased projection intraoperatively in order to compensate for the expected decline; however, this approach may also introduce variability, as the extent of postoperative change cannot be precisely predicted and may lead to overprojection if the anticipated reduction does not occur [19]. In the PTST, the observed decrease in projection was primarily confined to the early healing phase, while later follow-up intervals demonstrated smaller changes over time. This pattern suggests that projection changes become less pronounced following the initial postoperative period.

Previous studies evaluating structural rhinoplasty techniques such as septal extension grafts (SEG), columellar strut grafts (CSG), and tongue-in-groove (TIG) approaches have similarly demonstrated that postoperative decreases in nasal tip rotation and projection occur predominantly during the early healing period, followed by a tendency toward relative stabilization over time [6,20,21,22]. Comparative studies and recent systematic reviews have further suggested that SEG techniques may provide more consistent long-term maintenance of nasal tip rotation and projection than conventional CSG approaches, particularly with respect to rotational stability [17,23]. Similarly, Çelikal et al. [24] reported that projection and rotation loss following membranous TIG rhinoplasty was most pronounced during the early postoperative period, whereas later follow-up intervals demonstrated smaller temporal changes. The temporal pattern observed in the present study appears broadly consistent with these previously reported postoperative healing dynamics. However, unlike most previous studies that primarily focused on comparative graft outcomes or static postoperative measurements, the present study specifically evaluated interval-based temporal changes in nasal tip rotation and projection following PTST. Nevertheless, direct comparison between PTST and established structural rhinoplasty techniques remains limited because of differences in surgical methods, patient populations, measurement protocols, and study design.

Previous studies have suggested that postoperative reduction in nasal tip rotation and projection may be more pronounced in some patients with thick skin. This phenomenon has been attributed to the greater soft tissue volume and skin weight in these individuals, which may increase the downward forces acting on the nasal tip [25,26]. For this reason, surgeons may tend to create a greater degree of intraoperative over-rotation and projection in patients with thick skin in anticipation of increased postoperative reduction in rotation and projection [27,28]. However, this approach may also introduce variability, as the extent of postoperative change cannot be precisely predicted and may result in an over-rotated and/or overprojected appearance if the anticipated reduction does not occur. In the present study, skin type was not significantly associated with changes in rotation or projection during the later postoperative follow-up period. Although these findings should be interpreted cautiously, given the non-comparative design of the study, they may suggest that postoperative temporal changes observed with PTST are not markedly influenced by skin type during later follow-up intervals. From a clinical perspective, this observation may support a more controlled approach to intraoperative rotation and projection adjustment across different skin types. Nevertheless, further comparative studies with larger and more balanced cohorts are needed to clarify the influence of skin thickness on postoperative tip dynamics following PTST.

The temporal pattern observed with PTST may be related to its integrated structural design rather than to any single component alone. The asymmetric pentagonal configuration of the graft, together with its midline positioning at the anterior septal angle, was designed to provide structural support to the nasal tip complex. In addition, the use of multi-point fixation, including loop sutures and multiple interface sutures, allows close adaptation between the graft and septum while minimizing interfacial dead space. Integration of the graft with the domal complex may also contribute to coordinated support between the septum, graft, and lower lateral cartilages. From a theoretical perspective, this configuration may help distribute mechanical forces across the tip-supporting structures and may contribute to resistance against postoperative deformation. However, these potential mechanisms were not directly evaluated in the present study and should therefore be interpreted cautiously. Within this context, PTST should be regarded as a structural modification within the broader spectrum of established tip support techniques rather than as a completely independent alternative concept. Further comparative and biomechanical studies are required to clarify the structural behavior and postoperative behavior over time of PTST.

Although no major complications were observed in the present series, several technical considerations remain important for optimizing surgical outcomes. Precise graft sizing, stable fixation, and careful intraoperative adjustment of tip rotation are essential to minimize the risk of asymmetry, excessive projection, over-rotation, or postoperative tip stiffness. In addition, minor postoperative changes related to scar remodeling and soft tissue adaptation may still occur during the healing process. Future technical refinements and studies with larger patient populations and longer follow-up durations may further improve the reproducibility and stability of the technique over time.

This study has several limitations that should be considered. First, its retrospective and non-comparative design may introduce selection bias and limit the ability to establish causal relationships or determine superiority relative to other established techniques. Second, the study was conducted at a single center, and the majority of patients were female, which may limit the generalizability of the findings. In addition, nasal tip rotation and projection were assessed using standardized photographic analysis, which, although widely accepted, may be subject to a certain degree of measurement variability. Furthermore, the follow-up period was limited to 12 months, and longer-term postoperative changes beyond this period could not be evaluated. Functional outcomes and validated patient-reported outcome measures were not included in the analysis, limiting assessment of patient satisfaction and the broader clinical impact of the technique from the patient perspective. Finally, the absence of a control group prevented direct comparison of PTST with other commonly used structural rhinoplasty techniques such as septal extension grafts or columellar strut grafts. Future prospective comparative studies with longer follow-up periods and patient-reported outcome measures are needed to further clarify the clinical behavior of PTST over extended follow-up periods.

## 5. Conclusions

In conclusion, PTST demonstrated a temporal pattern of nasal tip behavior following rhinoplasty characterized by greater early postoperative changes followed by smaller interval changes during later follow-up. The observed temporal pattern may reflect continued structural support during the postoperative healing period. In addition, postoperative temporal changes appeared not to be markedly influenced by skin type during later follow-up intervals within this cohort. Although these findings should be interpreted within the limitations of a retrospective non-comparative study, the present results suggest that PTST may represent a useful structural approach for maintaining nasal tip rotation and projection following rhinoplasty. Further prospective comparative studies with longer follow-up periods are needed to better define its clinical behavior over extended follow-up periods and comparative performance relative to established techniques.

## Figures and Tables

**Table 1:** Demographic characteristics of the study population (*n* = 84).

Variable	
Age (years), Mean ± SD	28.0 ± 5.98
Gender, *n* (%)	
Male	4 (4.8%)
Female	80 (95.2%)
Skin Type, *n* (%)	
Thin	15 (17.9%)
Thick	33 (39.3%)
Normal	36 (42.9%)

Note. SD, standard deviation.

**Table 2:** Changes in nasolabial angle across postoperative follow-up intervals (*n* = 84).

Time Interval	Max Difference	Min Difference	Mean Difference	*p* Value
0–6 months	8.00	1.00	4.49 ± 1.31	<0.001
6–12 months	4.00	2.00	2.70 ± 0.53	<0.001
0–12 months	10.00	3.00	7.19 ± 1.44	<0.001

Note. Friedman test with Durbin–Conover post hoc pairwise comparisons. Values represent the magnitude of decrease between the specified time points.

**Table 3:** Changes in the Goode ratio across postoperative follow-up intervals (*n* = 84).

Time Interval	Max Difference	Min Difference	Mean Difference	*p* Value
0–6 months	0.042	0.001	0.019 ± 0.010	<0.001
6–12 months	0.008	0.000	0.003 ± 0.002	<0.001
0–12 months	0.044	0.003	0.022 ± 0.010	<0.001

Note. Friedman test with Durbin–Conover post hoc pairwise comparisons. Values represent the magnitude of decrease between the specified time points.

**Table 4:** Comparison of change magnitudes in nasal tip rotation and projection across postoperative intervals (*n* = 84).

Parameter	0–6 Months Difference (Mean ± SD)	6–12 Months Difference (Mean ± SD)	*p* Value *
Nasolabial Angle Change (°)	4.49 ± 1.31	2.70 ± 0.53	<0.001
Goode Ratio Change	0.019 ± 0.010	0.003 ± 0.002	<0.001

Note. * Friedman test with Durbin–Conover post hoc pairwise comparisons. SD: Standard Deviation.

**Table 5:** Multiple linear regression analysis of demographic predictors for nasolabial angle change between 6 and 12 months (*n* = 84).

Predictor	*β*	95% CI	*p* Value
Age	−0.106	−0.327, 0.116	0.346
Skin type: thick vs. thin	0.051	−0.577, 0.680	0.871
Skin type: normal vs. thin	0.065	−0.554, 0.685	0.835

Note. R = 0.109, R^2^ = 0.012, Adjusted R^2^ = −0.025, F(3, 80) = 0.320, *p* = 0.811. Reference category: thin (skin type). β, standardized regression coefficient; CI, confidence interval.

**Table 6:** Multiple linear regression analysis of demographic predictors for Goode ratio change between 6 and 12 months (*n* = 84).

Predictor	*β*	95% CI	*p* Value
Age	−0.002	−0.224, 0.220	0.985
Skin type: thick vs. thin	0.238	−0.392, 0.867	0.455
Skin type: normal vs. thin	0.206	−0.414, 0.827	0.510

Note. R = 0.087, R^2^ = 0.008, Adjusted R^2^ = −0.030, F(3, 80) = 0.201, *p* = 0.895. Reference category: thin (skin type). β, standardized regression coefficient; CI, confidence interval.

## Data Availability

The data presented in this study are available on request from the corresponding author.
